# A Comparative Study Between Single-Incision Laparoscopic Appendicectomy Using Conventional Instruments and Glove-Port (SILACIG) and Conventional Multiport Laparoscopic Appendicectomy (CMLA)

**DOI:** 10.7759/cureus.11257

**Published:** 2020-10-30

**Authors:** Ahamed Muneef, Uday Kumbhar, Chellappa Vijayakumar, Oseen Shaikh

**Affiliations:** 1 Surgery, Jawaharlal Institute of Postgraduate Medical Education and Research (JIPMER), Puducherry, IND

**Keywords:** single-incision laparoscopy, appendicectomy, glove-port

## Abstract

Introduction

Appendicectomy is the most common surgical procedure. Conventional laparoscopic appendicectomy being time-tested, attempts were made to make it less invasive. Single-incision laparoscopic appendicectomy is the most recent trend. The present study is conducted with the aim to compare surgical outcomes between single-incision laparoscopic appendicectomy using conventional instruments and glove-port (SILACIG) with conventional multiport laparoscopic appendicectomy (CMLA).

Materials and methods

A total of 80 patients with appendicitis were recruited and underwent SILACIG (n=40) and CMLA (n=40). They were monitored for operative time, time of oral intake, pain on the second postoperative day, day of discharge, return to work, and scar size after two months.

Results

There was no significant difference between SILACIG and CMLA in terms of the time of oral intake, day of discharge, and return to work. Operative time was significantly more in the SILACIG group as compared to CMLA. Pain on the second postoperative day was less than CMLA, and the size of the operative scar was significantly smaller than 2 cm in the SILACIG group as compared to the CMLA group.

Conclusion

SILACIG is a feasible, safe, and cost-effective technique. It is comparable with CMLA in terms of preoperative diagnosis, postoperative oral intake, hospitalization period, and return to work. It shows less pain on the second postoperative day and cosmetic benefit but requires more operative time than CMLA.

## Introduction

Appendicitis is the commonly encountered condition in surgical practice [1-2]. Surgery is the definitive treatment. Open surgical techniques are time-tested, followed by laparoscopy. In the laparoscopy, attempts were made to make it less invasive than the conventional multi-port one [3]. Examples are two-port, single-port [4], single-incision multi-port [5-6], natural orifice transluminal endoscopic surgeries (NOTES) [7-9], etc. There are various modifications of single-incision laparoscopic appendicectomy with merits and demerits of it over the conventional technique. The present study is conducted with the aim to compare surgical outcomes between single-incision laparoscopic appendicectomy using conventional instruments and glove-port (SILACIG) with conventional multi-port laparoscopic appendicectomy (CMLA).

## Materials and methods

The present study is an open-labeled comparative interventional study conducted in the department of general surgery at a tertiary-care teaching hospital in South India for 18 months. It started after obtaining institutional ethics committee approval. A total of 80 patients diagnosed with appendicitis were included in this study. The sample size was determined based on a pilot study in which the prevalence of usage of SILACIG was measured as 25%. We calculated a minimum sample size of 72 patients. The final sample selected was 40 patients in each group, assuming a type 1 error (two-tailed) of 0.05 and a margin of error of 10%. All the patients diagnosed with acute and chronic appendicitis by clinical and radiographic methods between 12 and 65 years of age without comorbid conditions (American Society of Anesthesiology Grades I and II) were included in the study. Patients of appendicitis with pregnancy, morbid obesity, multiple previous abdominal surgeries, and uncontrolled medical conditions (ischaemic heart disease, coagulopathy, uncontrolled hypertension, and diabetes) were excluded from the study. After taking informed consent, patients were assigned to the CMLA and SILACIG groups in a 1:1 ratio by alternate allocation; even-numbered patients were treated by CMLA (n = 40) and odd-numbered were treated by SILACIG (n = 40).

Surgical procedure

All the patients were operated on by using a standard laparoscopy set under general inhalational anesthesia with endotracheal intubation.

**Figure 1:**
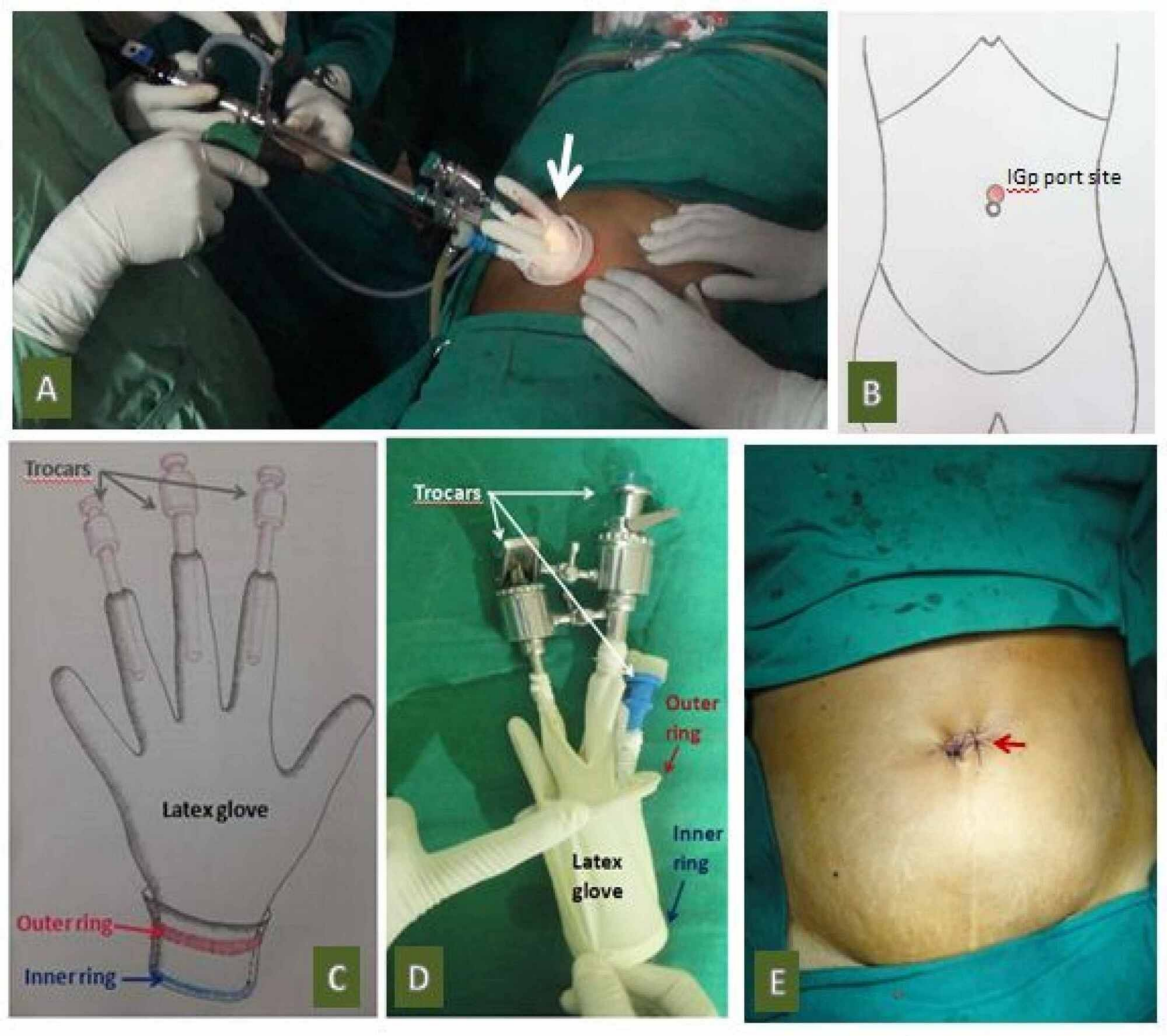
Images of SILACIG showing: A: Intraoperative external view of the glove-port (arrow); B: Schematic diagram of the port placement; C: Schematic diagram of glove-port; D: Prepared glove-port ready for insertion; and E: Immediate postoperative port-site SILACIG: single-incision laparoscopic appendicectomy using conventional instruments and glove-port

A) SILACIG surgical technique (Video 1): The operating surgeon stood to the patients left. The camera assistant sat on the right, and the staff nurse stood to the left of the operating surgeon. The monitor was on the right side of the patient; a single supra-umbilical curved incision measuring 2.0 cm was given. The umbilical tube was dissected. A vertical incision was made over the tube to enter the peritoneal cavity. The incision over the tube measured approximately 1.5 to 2.0 cms, and the indigenous In-house made glove port (IGp) was introduced into the peritoneal cavity through it (Figure 1). The pneumoperitoneum was created. The entire peritoneal cavity was visualized to confirm the diagnosis and note the position of the appendix. The appendix was caught with Babcock forceps and the mesoappendix cauterized using bipolar diathermy. The appendix base was secured with three catgut Endoloops (J&J Medical Devices, Mexico) and cut between two proximal and one distal loop. The appendix was retrieved by placing it in one of the glove's unused fingers, and that glove-finger isolated by tying it with thread at the base. The specimen was removed by cutting the tip of the glove and sent for histopathological examination. The glove-port was removed after deflating the pneumoperitoneum. The umbilical tube incision was sutured using 1-0 vicryl. The skin was sutured with 2-0 Ethilon (Ethicon Inc., Somerville, New Jersey) (Figure 1E). The details of the preparation of the glove-port and the entire procedure are already published [10].

**Video 1:** Operative procedure of SILACIG SILACIG: single-incision laparoscopic appendicectomy using conventional instruments and glove-port

**Figure 2:**
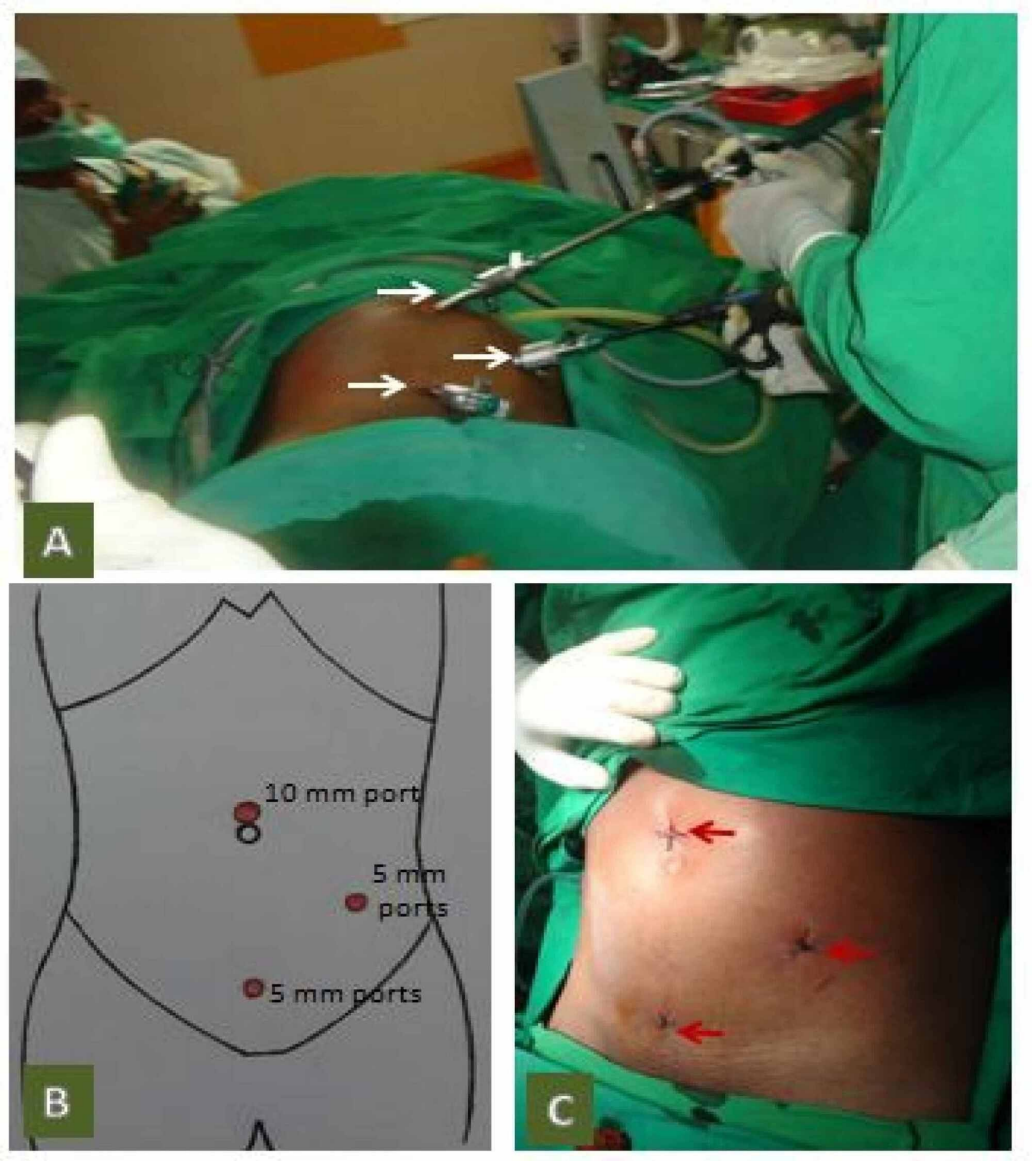
Images of CMLA showing: A: Intraoperative external view with three ports (arrows); B: Schematic diagram of the port placement; and C: Immediate postoperative port-sites (arrows) CMLA: conventional multiport laparoscopic appendicectomy

B) CMLA technique: The position of the patient, operating team, and monitor trolley were the same as described for SILACIG. One trocar was placed in the umbilicus (10 mm), the second trocar (5 mm) in the supra-pubic region, and the third trocar (5 mm) in the left iliac fossa (Figures 2A-2B). Initially, the abdomen was thoroughly explored to exclude other pathology. The appendix was identified by following the anterior taenia to its base. The appendix was caught with Babcock forceps, and the mesoappendix cauterized using bipolar diathermy. The appendix's base was secured with three catgut Endoloops and cut between two proximal and one distal loop. The appendix was retrieved through the 10 mm trocar. The base of the appendix and the mesoappendix were examined for hemostasis. Trocars were removed under direct vision after deflating the pneumoperitoneum and the port sites were closed using 2-0 polypropylene (Figure 2C).

Postoperative care

Patients were given standard postoperative care in the form of intravenous antibiotics (cefotaxime), analgesic (injection paracetamol 1 gm intravenous infusion twice daily), and adequate intravenous fluids.

Study variables

For each patient, the following variables were recorded in the preformed proforma.

Operative Time

The amount of time taken in hours from the insertion of the first trocar to closure of the port site in conventional laparoscopic appendicectomy and from the insertion of IGp to port closure in SILACIG. 

Pain on the 2nd Postoperative Day

Pain monitored according to the visual analog scale (VAS) from 1-10.

Oral Fluid Intake Time

The time taken in hours for starting oral fluid postoperatively.

Day of Discharge

The number of days patients admitted postoperatively. 

Return to Work

The calendar day the patient returns to work postoperatively.

Cosmesis

Size of the scar measured in centimeters two months after surgery. 

Statistical analysis

Descriptive statistics were done for all data, and suitable statistical tests of comparison done. Continuous variables were analyzed with the unpaired t-test and categorical variables were analyzed with the chi-square test and Fisher's exact test. Statistical significance was taken as p < 0.05. The data were analyzed using EpiInfo software (7.1.0.6 version; Center for Disease Control, USA) and Microsoft Excel 2010 (Microsoft Corporation, Redmond, WA).

## Results

In the present study, the patient's minimum age was 12 years, whereas the maximum age was 63 years. The mean age in the SILACIG group was 27.48 years, and in the CMLA group, it was 26.6 years. There was a male preponderance with a male:female ratio of 2.3:1 and 2:1 in the SILACIG and CMLA groups. In the SILACIG group, 47.5% and in the CMLA group, 25% of patients presented with acute appendicitis. In acute cases, the procedure was performed on an emergency basis, and the rest of the cases were operated electively. The details of perioperative variables are depicted in Table 1.

**Table 1:** Comparison of perioperative variables between SILACIG and CMLA * Statistically significant with p-value <0.05 SILACIG: single-incision laparoscopic appendicectomy using conventional instruments and glove-port; CMLA: conventional multiport laparoscopic appendicectomy

S. No.	Variables	SILACIG	CMLA	Chi-square test	Degree of freedom	p-value
1	Operative time			21	2	0.000*
	<1 hr	6 (15%)	26 (65%)			
	≥1 hr	34 (85%)	14 (35%)			
2	Pain on 2^nd ^postoperative day			27.9	5	0.000*
	VAS ≤ 4	34 (85%)	24 (60%)			
	VAS > 5	6 (15%)	16 (40%)			
3	Time of oral intake			6.18	3	0.103
	≤ 24 hrs	38 (95%)	33 (83.5%)			
	>24 hrs	2 (5%)	7 (17.5%)			
4	Day of discharge			3.11	3	0.375
	3^rd^ POD	31 (77.5%)	24 (60%)			
	≥ 4^th^ POD	9 (22.5%)	16 (40%)			
5	Day of return to work			1.67	2	0.434
	3^rd^ week	35 (87.5%)	31 (77.5%)			
	≥ 4^th^ week	5 (12.5%)	9 (22.5%)			
6	Scar size after 2 months			80	1	0.000*
	≤ 2 cm	40 (100%)	0 (0%)			
	> 2 cm	0 (0%)	40 (100%)			

**Figure 3:**
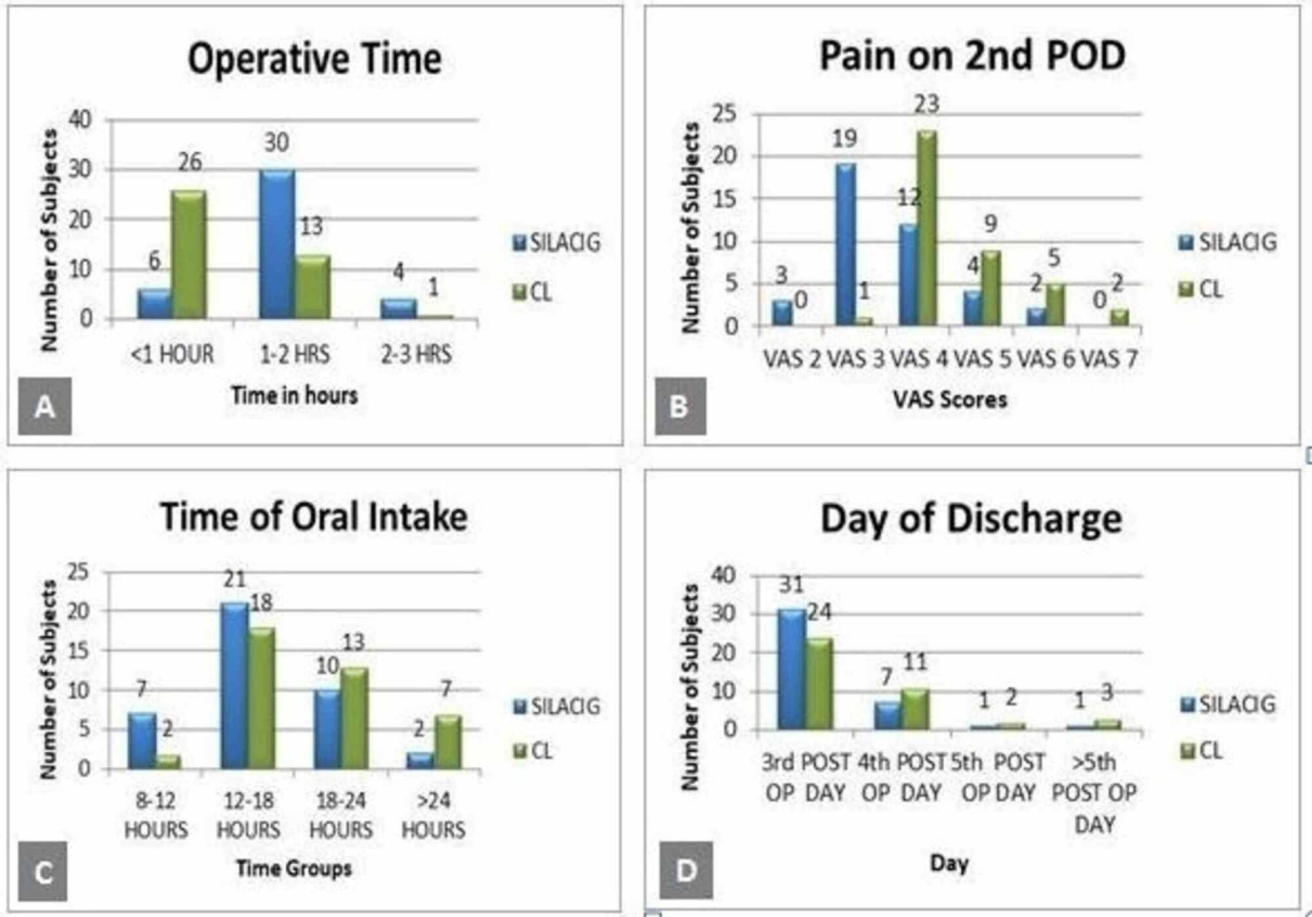
Bar diagrams showing the comparison of SILACIG and CMLA with respect to: A: Operative time; B: Pain on second postoperative day; C: Time of oral intake; and D: Day of discharge SILACIG: single-incision laparoscopic appendicectomy using conventional instruments and glove-port; CMLA: conventional multiport laparoscopic appendicectomy

**Figure 4:**
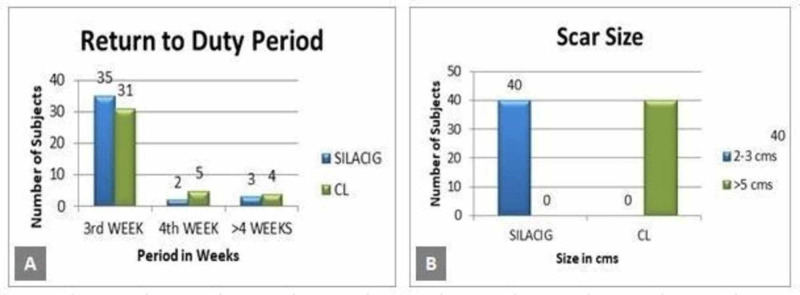
Bar diagrams showing the comparison of SILACIG and CMLA with respect to: A: Return to duty period and B: Postoperative scar size after two months of surgery SILACIG: single-incision laparoscopic appendicectomy using conventional instruments and glove-port; CMLA: conventional multiport laparoscopic appendicectomy

Operative time was significantly more in the SILACIG group as compared to the CIMLA group (Figure 3A). Most of the SILACIG group patients had less pain on the second postoperative day as compared to the CMLA group (Figure 3B). More than 80% of the patients tolerated oral feed in both groups within 24 hours (Figure 3C). In both groups, most of the patients returned for work at the end of the third week (Figure 4A). On the operative scar assessment two months postoperatively, it found that all the patients in the SILACIG group had scar size less than 2 cm, whereas it was more than 2 cm in all patients in the CIMLA group (Figure 4B).

## Discussion

The relationship between endosurgical approaches has been the subject of much debate in recent years. Appendicectomy is a standard procedure and thus lends itself suitable for a comparison of surgical techniques [11]. Some clinicians believe single-incision laparoscopic surgery may be embraced over other novel surgical innovations, such as NOTES. In light of this fact, it is based on the current practice of incision in proximity to the umbilicus laparoscopic instruments and camera providing access and view.

In the present study, there was no statistically significant difference between the two groups in terms of age, gender, pre-operative diagnosis, and presentation (emergency or elective). Thus both groups were comparable. This study indicated that although SILACIG was associated with a longer operative time, it had less pain on the second postoperative day and better cosmetic satisfaction than CMLA. No significant differences were found in the time of oral intake, length of hospital stay, and return to work between the two procedures.

Operative time for SILACIG is longer than that for CMLA. The reasons may be the collision of instruments and the telescope due to the absence of triangulation as happens in other single-incision surgeries. Another reason for the consumption of more time was the chance of glove tear during the insertion and removal of instruments, as occurred in three of our cases, where it required changing of the port. This time can be reduced by keeping another standby IGp ready. Added to that, performing tasks using the SILACIG technique is more technically challenging than when using a standard laparoscopic technique, even for surgeons with previous single incision laparoscopic surgical experience. Performing SILACIG requires experience in laparoscopic surgery, and a certain number of cases must be performed to overcome the learning curve. A retrospective study by Lee et al. reported that the operation time tended to shorten when the surgeon gained more experience and accumulated cases [12]. A separate study by Perez et al. reported that in the first 25 patients enrolled, the difference in operative time was significantly higher (49.31 Vs. 33.50 min, p = 0.049), and this difference decreased in a subsequent group of 25 patients (44.08 Vs. 36.00 min, p = 0.123) [13]. In our study also, the time taken initially was longer than one hour, and it decreased to less than one hour in later cases. However, a systematic review by Gill RS et al. found no operative time difference between the single-incision and multiport laparoscopic appendicectomy groups [14].

Pain on the second postoperative day was statistically significant. Although its clinical significance is doubtful as pain is a subjective parameter, and its varied perception by each subject, we cannot confirm a clinically significant difference between the two groups. Moreover, the potential of SILACIG as an attractive clinical option to achieve pauci-traumatic access surgery needs additional evaluation to reduce the surgical trauma further. Teoh et al. [15] and Lee et al. [12] observed that there was no difference in the postoperative pain scores between the single-incision and multiport laparoscopic appendicectomy. However, in Frutos' trial, less pain was found in the single-incision laparoscopic appendicectomy group as compared to conventional laparoscopic appendicectomy (SILA/CLA: 2.76 ± 1.64/3.78 ± 1.76, p < 0.001). The theoretical explanation for less pain in single incision surgeries is fewer fascial injuries than in multiport modality [16].

There is no technique-related difference between the two groups in terms of postoperative oral feeding. In their study, Kyung et al. compared single-incision laparoscopic appendicectomy with one port and three-port appendicectomy. There was no procedure-related statistically significant difference in the first passage of flatus and start of diet [17].

The day of discharge had no difference in both the groups. Kyung et al. state that the hospitalization periods were 6.8 ± 1.8 days, 6.2 ± 1.5 days, and 6.4 ± 1.5 days for single-incision laparoscopic appendectomy, one-port single incision laparoscopic surgery, and conventional three-port laparoscopic appendectomy, respectively [17]. The interval to the first gas out after surgery, the start of the diet, and the hospitalization period showed no statistically significant difference.

The two procedures SILACIG and CMLA do not differ concerning the patient's return to work postoperatively even though the pain morbidity on the second postoperative day varies. Teoh AY et al. state that no differences in the quality-of-life assessments were present at two weeks after operation [15].

The scar size produced by SILACIG is significantly less than 2 cm when compared to the conventional technique. This study has highlighted cosmetic satisfaction as the significant benefit of SILACIG over CMLA. This so-called "scarless" procedure meets the demand of expecting to conceal the surgical history of patients, especially in young females. Kyung et al. also reported a reduced scar in the single incision as compared to the three-port laparoscopic appendicectomy group [17]. Teoh et al. concluded that wound cosmesis and satisfaction scores were better in the laparoendoscopic single-site access surgery (LESS) group compared to the conventional laparoscopic technique [15]. Yu-Long et al., in their meta-analysis, concluded that single-incision laparoscopic appendicectomy has the benefit of cosmetic satisfaction as compared to conventional appendicectomy [18].

SILACIG also has various advantages: low cost, readily available, easy to prepare IGp, and reproducible. Various methods of specimen retrieval, such as Endobag retrieval and Fisherman technique, are described. We used the simple method of retrieving it through one of the unused glove fingers [19-21]. Retrieval of the specimen without touching the wound reduces the chance of port site infection.

Limitations of the study: It is a single-center, non-randomized study without blinding. Pain on the second postoperative day is low in the SILACIG group, which needs further evaluation before coming to its clinical significance, as it is subjective.

## Conclusions

The present study concludes that SILACIG is a feasible, safe, and cost-effective technique. It is comparable with CMLA in terms of preoperative diagnosis, postoperative oral intake, hospitalization period, and return to work. The operative time in SILACIG is significantly more as compared to CMLA, which can reduce with experience. Pain on the second postoperative day is statistically less than in CMLA but needs further evaluation before coming to its clinical significance. SILACIG has cosmetic benefits over CMLA, both statistically as well as clinically.
